# Paraspinal muscles and gluteus medius fat infiltration are both associated with lumbar disc herniation

**DOI:** 10.1186/s13244-025-02064-9

**Published:** 2025-08-14

**Authors:** Jianping Wang, Zhenhua Zhao, Ding Liang, Xiao Xu, Dingbo Shu

**Affiliations:** 1 Department of Radiology, Shaoxing People’s Hospital, Shaoxing, China; 2 Key Laboratory of Functional Molecular Imaging of Tumor and Interventional Diagnosis and Treatment of Shaoxing City, Shaoxing, China

**Keywords:** Lumbar disc herniation, Paraspinal muscle, Gluteus medius, Degeneration, Proton density fat fraction

## Abstract

**Objectives:**

To quantitatively assess the degeneration of paraspinal and pelvic muscles in patients with lumbar disc herniation (LDH) using MRI q-Dixon and to explore their potential association with LDH.

**Materials and methods:**

The cross-sectional area (CSA) and proton density fat fraction (PDFF) of the multifidus (MF), erector spinae (ES), psoas major (PM), and gluteus medius (GM), as well as the vertebral bone marrow fat fraction (BMFF), lumbar lordosis (LL), and sacral slope (SS), were measured in both LDH and control groups.

**Results:**

A total of 85 LDH patients and 48 controls were included. No significant differences in CSA were observed between groups (all *p* > 0.05). However, PDFF values were significantly higher in the LDH group for all muscles compared to controls (all *p* < 0.05). After adjustment, the PDFF differences in MF_L4/5_, ES_L4/5_, PM_L3/4_, and PM_L4/5_ remained statistically significant (*p* < 0.05). Although bilateral muscle asymmetry was observed in several muscles, these differences were no longer significant after adjustment (*p* > 0.05). No significant differences in VBFF, LL, or SS were found (all *p* > 0.05).

**Conclusions:**

LDH patients exhibit increased fat infiltration in the MF, ES, PM, and GM compared to controls, suggesting a potential association between pelvic and paraspinal muscle degeneration and LDH.

**Critical relevance statement:**

The MRI q-Dixon technique effectively and noninvasively detects early degeneration of the paraspinal muscles and GM, indicating their potential association with LDH and enabling more targeted rehabilitation strategies.

**Key Points:**

- Spine and muscle changes affect LDH, but their relationship remains unclear.
- Fat infiltration is more sensitive than CSA for detecting muscle degeneration.
- LDH patients exhibit greater paraspinal muscle and GM degeneration.

**Graphical Abstract:**

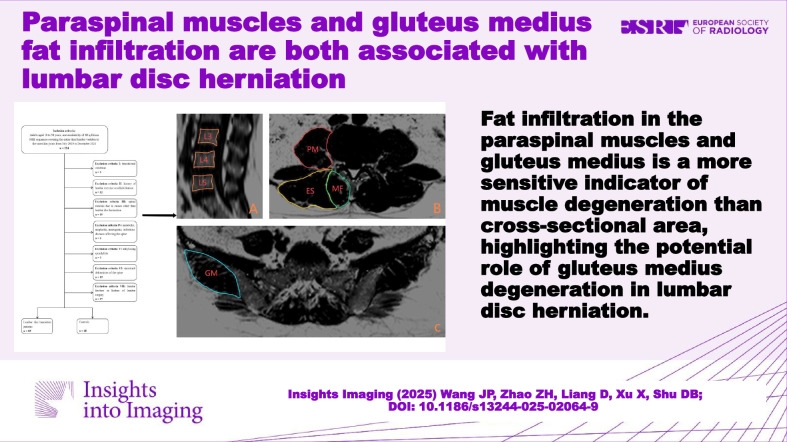

## Introduction

Lumbar disc herniation (LDH) is a common degenerative disease that can lead to low back pain, radiating pain in the lower extremities, and limited mobility, and may progress to spinal instability, facet joint degeneration, and spinal stenosis [1–3]. The paraspinal muscles, including the multifidus (MF), erector spinae (ES), and psoas major (PM), are the primary components of the trunk musculature and maintain spinal stability and function [4, 5]. Recent studies have emphasised the importance of paraspinal muscles in maintaining lumbar spine health, with atrophy, fatty infiltration, and fibre type transformation of these muscles being closely associated with LDH [6–8]. Dysfunction or atrophy of these muscles compromises lumbar stability, leading to uneven pressure distribution on the lumbar discs, which may subsequently lead to damage of adjacent spinal structures [2, 9]. These alterations may contribute to the onset or progression of low back pain and spinal dysfunction [10]. In addition to paraspinal muscle alterations, sagittal spinal alignment and changes in vertebral composition are also closely linked to lumbar disc health. Increased bone marrow fat content within the vertebra has been shown to impair nutrient supply to disc cells, potentially accelerating disc degeneration [11, 12]. Furthermore, reduced lumbar lordosis (LL) and sacral slope (SS) can elevate intradiscal pressure, contributing to disc degeneration [13, 14]. Additionally, the pelvis is closely linked to the spine, with the gluteus medius (GM) providing lateral stability to the hips and spine, while interacting with the paraspinal muscles to stabilise the pelvis [15, 16]. Dysfunction of the pelvic muscles may further compromise spinal health.

Magnetic resonance imaging (MRI), with its high resolution, superior soft-tissue contrast, and capacity to visualise deep tissue anatomy, is an effective tool for the noninvasive evaluation of lower lumbar and pelvic muscles [11, 17–19]. However, conventional T1- and T2-weighted MRI sequences may not yield quantitative or objective information regarding paraspinal muscle components [20]. The q-Dixon technique, based on advanced chemical shift-encoded MRI, generates a fat fraction map and calculates proton density fat fraction (PDFF) from the acquired data [21–23]. The reliability of this technique in detecting intramuscular lipids is comparable to that of muscle biopsy, offering a broader examination range and superior repeatability [24, 25]. In the assessment of vertebral bone marrow fat fraction (BMFF), Lee et al demonstrated that the q-Dixon technique closely aligns with magnetic resonance spectroscopy for the quantitative evaluation of vertebral BMFF [26].

Therefore, the aim of this study was to emphasise the integrity of the paraspinal and pelvic muscles by measuring the cross-sectional area (CSA) and PDFF of the MF, ES, PM, and GM, as well as the vertebral BMFF, LL and SS using the q-Dixon based MRI technique, to explore factors associated with LDH.

## Materials and methods

### Subjects

This retrospective study was approved by our institutional review board (2025-SRP-056-Y-01), which waived the requirement for patient consent or written informed consent for the review of medical records or images. Subjects who underwent lumbar MRI at our institution between July 2023 and December 2024 were evaluated and screened. Inclusion criteria for the study were as follows: (1) adults aged 18–50 years, and (2) availability of 3D q-Dixon MRI sequences covering the entire third lumbar vertebra to the sacroiliac joints. For the LDH group, inclusion required the presence of one or more disc herniations at the L3/4–L5/S1 levels, including bulging, protrusion, extrusion, or sequestration [27]. Exclusion criteria were: (1) transitional vertebrae (vertebrae exhibiting characteristics of both lumbar and sacral vertebrae) [28]; (2) history of lumbar exercise or rehabilitation; (3) spinal stenosis due to causes other than LDH; (4) presence of metabolic, neoplastic, neurogenic, or infectious diseases affecting the spine; (5) ankylosing spondylitis; (6) structural deformities of the spine (e.g., scoliosis, kyphosis); and (7) lumbar fracture or history of lumbar surgery. Demographic data, including age, sex, and body mass index (BMI), were collected for all participants.

### MRI protocol

Subjects were examined in the supine position using a 1.5-T MRI scanner (Magnetom Aera, Siemens Healthcare) and an 8-channel phased-array spine coil. The examination sequence involved in this study was a sagittal T1-weighted fast spin echo sequence with the following parameters: TR = 540 ms, TE = 9 ms, FOV = 280 × 280 mm, thickness = 4 mm, average = 2, and scanning time of 1 min and 51 s; sagittal T2-weighted fast spin echo sequence with the following parameters: TR = 2400 ms, TE = 91 ms, FOV = 280 × 280 mm, thickness = 4 mm, average = 2, and scanning time of 1 min and 46 s; and transverse 3D q-Dixon sequence with the following parameters: TR = 15.81 ms, TE = 2.38, 4.76, 7.14, 9.52, 11.90, 14.28 ms, FOV = 320 × 240 mm, thickness = 3 mm, average = 2, and scanning time of 40 s. Besides these three sequences, our institutional routine lumbar spine MRI protocol also includes sagittal fat-suppressed T2-weighted fast spin echo and transverse T2-weighted fast spin echo sequences.

### Image analysis

**Fig. 1:**
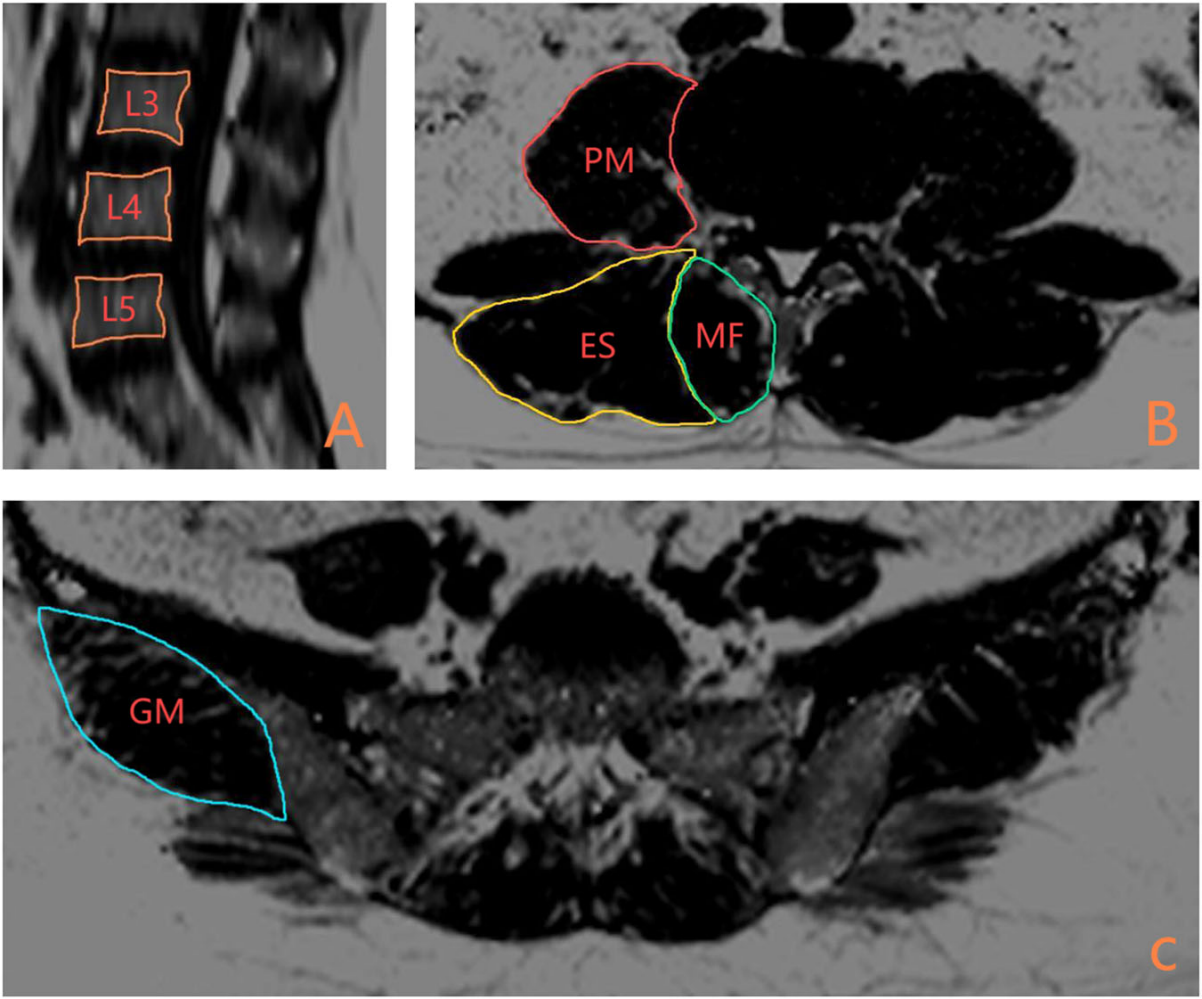
**A**–**C** As an example of image segmentation. **A** shows vertebral segmentation from L3 to L5. **B** shows muscle segmentation with the PM in red, the MF in green, and the ES in yellow, while **C** Highlights the GM in blue. PM, psoas major; MF, multifidus; ES, erector spinae; GM, gluteus medius

**Fig. 2:**
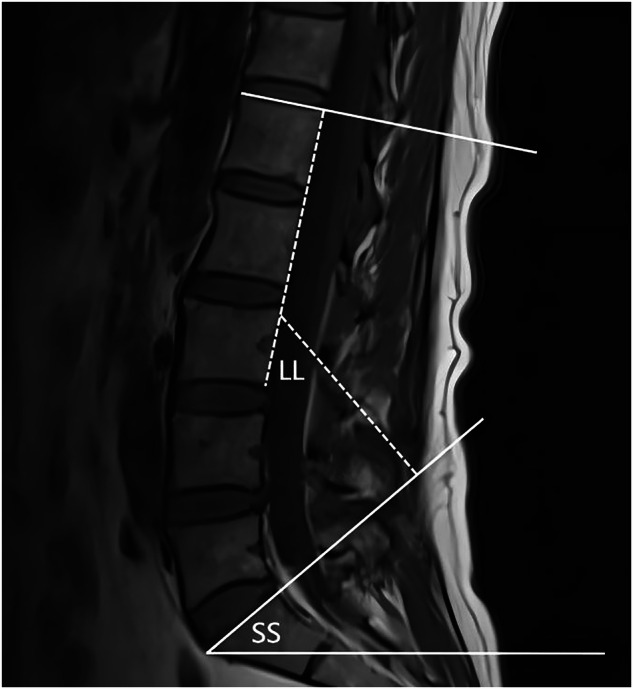
As shown in the figure, LL is defined as the angle between the superior endplates of L1 and S1, while SS is defined as the angle between the superior endplate of S1 and the horizontal line. LL, lumbar lordosis; SS, sacral slope

Image analysis was performed using ImageJ software (National Institutes of Health). The CSA and PDFF of the MF, ES, and PM were measured at the mid-disc level of the L3/4 and L4/5 lumbar discs on single-slice axial q-Dixon fat quantification images (Fig. 1B) [29–31]. For the GM, measurements were obtained from the most cranial slice of the axial q-Dixon fat quantification images where the sacroiliac joint was clearly visualised (Fig. 1C). Bilateral muscle measurements were independently recorded and subsequently either averaged or used to calculate asymmetry indices, depending on the analytic objective. Muscle asymmetry was assessed using both absolute differences and relative percentage differences, with the latter calculated using the formula: Asymmetry (%) = [(larger side − smaller side)/larger side] × 100. Fat quantification images obtained from the q-Dixon sequences were then reconstructed, followed by segmentation of the L3, L4, and L5 vertebral bodies at the median sagittal level to calculate the vertebral BMFF from the reconstructed images (Fig. 1A). LL and SS were measured on T1-weighted images within the Picture Archiving and Communication System at the clinical workstation (Fig. 2).

All MRI images were independently analysed by two radiologists (J.W. and D.S.) with 7 years and 6 years of experience in clinical diagnosis and spinal MRI, respectively. The final quantitative results were based on the average of the measurements from both experts. Prior to analysis, the radiologists standardised the segmentation method, and all documents were anonymised to ensure that patient demographic information remained confidential. To assess measurement consistency, inter-rater reliability was evaluated using the intraclass correlation coefficient (ICC).

### Statistics

All statistical analyses were conducted using SPSS software (version 26.0; IBM Corp) and Python (version 3.9). Asymmetry between the muscles was assessed by subtracting the CSA and PDFF of bilateral muscles and calculating the absolute value. The Shapiro–Wilk test was employed to determine the normality of the measurement data. Data conforming to a normal distribution were expressed as mean ± standard deviation, while non-normally distributed data were presented as median and interquartile range. Group differences were initially assessed using independent *t*-tests or the Mann*–*Whitney *U*-test. To control for potential confounding factors (age, sex, and BMI), multiple linear regression models were applied. In each analysis, the measured parameter served as the dependent variable, with the group indicator (LDH vs control) as the primary independent variable and age, sex, and BMI as covariates, in order to assess adjusted group differences. Significance level was set at *p* < 0.05. Receiver operating characteristic (ROC) curve analysis was performed to compare the diagnostic performance of CSA and PDFF for LDH across muscle groups. Areas under the curve (AUCs) were calculated, and DeLong’s test was used to assess differences in AUCs. To evaluate whether muscle degeneration differed by herniation severity, a subgroup analysis within the LDH group was conducted using the Kruskal–Wallis test. Based on a two-tailed independent *t*-test with an effect size of *d* = 0.6, an alpha level of 0.05, and a power of 0.8, the required sample size was calculated to be 90 participants (45 per group). Effect sizes were computed using Cohen’s *d*, with values of 0.2, 0.5, and 0.8 representing small, medium, and large effect sizes, respectively [32].

## Results

### Demographic characteristics

**Fig. 3:**
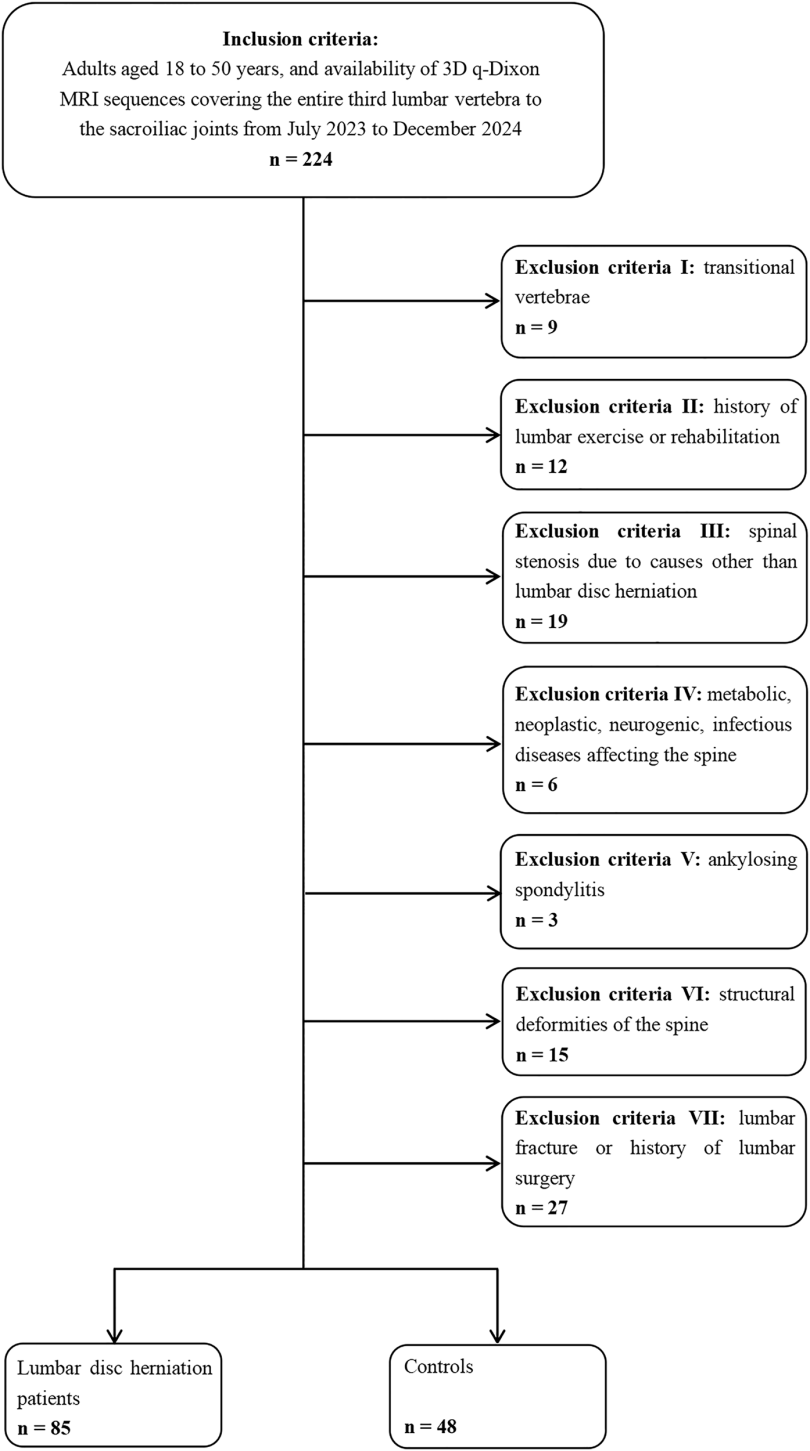
Flowchart of patient inclusion

A total of 85 eligible LDH patients (51.8% female) and 48 controls (60.4% female) were included in the analysis. The physical characteristics of all subjects are summarised in Table 1. Age and sex distribution were similar between the LDH and control groups (*p* > 0.05), while BMI was significantly higher in the LDH group (*p* < 0.001). The flowchart is shown in Fig. 3.

**Table 1:** Subjects’ characteristics

	LDH (*n* = 85)	Control (*n* = 48)	*p*-value
Age (year), mean (SD)	36.9 (8.1)	34.3 (8.3)	0.078
Female, n (%)	44 (51.8%)	29 (60.4%)	0.239
BMI (kg/m^2^), mean (SD)	24.2 (3.2)	21.8 (3.1)	< 0.001

*LDH* lumbar disc herniation, *BMI* body mass index, *SD* standard deviation

### Inter-rater reliability

The ICCs demonstrated excellent agreement between the two radiologists, with values of 0.986 for CSA, 0.975 for PDFF, and 0.997 for spinal parameters, including VBFF, LL, and SS.

### Muscle CSA and PDFF

There were no significant differences in the CSA of any muscles between the groups, including adjusted results (all *p* > 0.05, *d* = 0.18–0.36). In contrast, PDFF values for all muscles were higher in the LDH group compared to the control group (all *p* < 0.05, *d* = 0.37–0.86). The differences in PDFF for MF_L4/5_, ES_L4/5_, PM_L3/4_, and PM_L4/5_ remained statistically significant after adjustment (*p* < 0.05), while the difference in GM PDFF showed marginal significance (*p* = 0.08) (Table 2). Regression coefficients, *p*-values, and R² values were reported for each model assessing muscle PDFF (Supplementary Table 1). PDFF showed significantly higher AUC values than CSA in the PML3/4 (0.738 vs 0.576, *p* = 0.016) and PML4/5 (0.725 vs 0.572, *p* = 0.024). Detailed results are provided in Supplementary Table 2.

**Table 2:** Crude and adjusted analyses of muscle CSA and PDFF between the two groups

	LDH (*n* = 85), mean ± SD	Control (*n* = 48), mean ± SD	*d*	*p* Crude	*p* Adjusted
CSA (cm²)					
MF_L3/4_	641.8 ± 153.5	591.5 ± 123.9	0.36	0.054	0.785
MF_L4/5_	970.3 ± 184.5	930.5 ± 188.7	0.21	0.238	0.500
ES_L3/4_	1901.1 ± 497.9	1744.5 ± 562.8	0.29	0.099	0.759
ES_L4/5_	1358.8 ± 348.7	1295.5 ± 345.4	0.18	0.315	0.991
PM_L3/4_	1042.0 ± 371.7	954.3 ± 351.4	0.24	0.185	0.875
PM_L4/5_	1304.0 ± 399.4	1212.8 ± 384.6	0.23	0.202	0.993
GM	1531.1 ± 501.4	1360.9 ± 630.9	0.30	0.090	0.954
PDFF (%)					
MF_L3/4_	14.5 ± 5.7	12.5 ± 5.0	0.37	**0.047**	0.223
MF_L4/5_	16.2 ± 5.5	13.6 ± 4.7	0.51	**0.005**	**0.028**
ES_L3/4_	11.6 ± 4.6	8.9 ± 4.3	0.61	**0.001**	0.123
ES_L4/5_	19.4 ± 7.8	14.5 ± 7.0	0.66	**<** **0.001**	**0.031**
PM_L3/4_	9.5 ± 2.9	7.0 ± 2.9	0.86	**<** **0.001**	**0.011**
PM_L4/5_	10.5 ± 3.3	7.8 ± 3.3	0.82	**<** **0.001**	**0.038**
GM	15.0 ± 5.5	11.3 ± 4.5	0.74	**<** **0.001**	0.080

Values were calculated as the average of the parameters from both sides of the muscles

Bold values indicate *p* < 0.05

*CSA* cross-sectional area, *PDFF* proton density fat fraction, *LDH* lumbar disc herniation, *SD* standard deviation, *MF* multifidus, *ES* erector spinae, *PM* psoas major, *GM* gluteus medius

### Symmetry of bilateral muscle CSA and PDFF

The Mann–Whitney nonparametric *U*-test was applied, as the data did not follow a normal distribution (*p* < 0.05). In the crude analysis, muscle asymmetry was significant in CSA of the ES_L4/5_ (*p* = 0.009), PDFF of the PM_L4/5_ (*p* = 0.025), and PDFF of the GM (*p* = 0.029). However, these differences were no longer significant after adjustment (*p* > 0.05). No significant differences were observed in the remaining muscle parameters in either the crude or adjusted analyses (*p* > 0.05) (Table 3). In addition, when muscle asymmetry was calculated as a relative percentage difference, no statistically significant differences were observed between the LDH and control groups for any muscle parameter (all *p* > 0.05), except for the CSA of ES_L4/5_, which showed a crude *p*-value of 0.022 prior to adjustment (Table 4).

**Table 3:** Crude and adjusted analyses comparing muscle CSA and PDFF asymmetry between the two groups (absolute difference)

Variable	LDH (*n* = 85), median (IQR)	Control (*n* = 48), median (IQR)	*p* Crude	*p* Adjusted
CSA (cm²)				
MF_L3/4_	57.6 (23.6–105.3)	60.5 (27.0–94.4)	0.938	0.615
MF_L4/5_	64.6 (33.9–98.9)	60.5 (18.4–99.2)	0.648	0.632
ES_L3/4_	126.1 (44.2–238.7)	121.0 (57.0–179.7)	0.549	0.489
ES_L4/5_	127.4 (48.3–171.8)	71.3 (29.3–127.4)	**0.009**	0.081
PM_L3/4_	82.6 (30.1–145.3)	66.6 (31.5–108.3)	0.488	0.467
PM_L4/5_	75.5 (27.2–122.9)	82.9 (38.2–140.2)	0.593	0.953
GM	290.6 (151.7–599.7)	267.8 (123.7–424.8)	0.183	0.435
PDFF (%)				
MF_L3/4_	1.9 (0.8–4.0)	2.1 (1.1–3.5)	0.892	0.631
MF_L4/5_	2.5 (1.1–3.1)	2.0 (0.7–3.5)	0.339	0.638
ES_L3/4_	1.1 (0.6–2.1)	1.0 (0.4–1.5)	0.080	0.495
ES_L4/5_	2.1 (1.3–5.0)	1.7 (1.0–3.2)	0.056	0.354
PM_L3/4_	1.4 (0.6–2.5)	0.9 (0.3–1.9)	0.055	0.236
PM_L4/5_	1.9 (0.9–2.7)	1.5 (0.7–1.9)	**0.025**	0.174
GM	2.1 (9.1–4.7)	1.5 (0.6–3.0)	**0.029**	0.128

Values were calculated as the absolute difference between the corresponding muscle parameters on the left and right sides

Bold values indicate *p* < 0.05

*CSA* cross-sectional area, *PDFF* proton density fat fraction, *LDH* lumbar disc herniation, *IQR* interquartile range, *MF* multifidus, *ES* erector spinae, *PM* psoas major, *GM* gluteus medius

**Table 4:** Crude and adjusted analyses comparing muscle CSA and PDFF asymmetry between the two groups (percentage difference)

Variable	LDH (*n* = 85), median (IQR)	Control (*n* = 48), median (IQR)	*p* Crude	*p* Adjusted
CSA (%)				
MF_L3/4_	8.6 (3.7–15.0)	9.6 (4.7–16.7)	0.710	0.830
MF_L4/5_	6.6 (3.2–9.2)	6.7 (2.4–10.7)	0.965	0.563
ES_L3/4_	6.4 (2.6–13.3)	7.1 (3.6–10.8)	0.752	0.325
ES_L4/5_	8.2 (4.1–12.8)	6.3 (1.9–9.2)	**0.022**	0.067
PM_L3/4_	7.7 (3.1–12.3)	8.1 (3.7–13.6)	0.828	0.467
PM_L4/5_	5.5 (2.3–9.3)	6.1 (3.4–10.7)	0.336	0.625
GM	19.6 (9.6–34.6)	16.7 (11.7–28.3)	0.557	0.404
PDFF (%)				
MF_L3/4_	12.9 (7.1–20.7)	17.1 (10.3–24.3)	0.282	0.537
MF_L4/5_	13.7 (8.0–18.5)	14.9 (6.2–21.7)	0.809	0.738
ES_L3/4_	10.5 (6.8–17.9)	10.7 (4.4–20.6)	0.950	0.354
ES_L4/5_	12.9 (7.9–19.7)	13.2 (7.1–24.1)	0.734	0.636
PM_L3/4_	14.4 (7.0–22.3)	16.7 (6.8–25.5)	0.717	0.885
PM_L4/5_	17.5 (8.8–24.6)	18.0 (9.6–25.5)	0.611	0.878
GM	15.9 (6.4–27.5)	14.0 (5.9–26.5)	0.414	0.213

Values were calculated as the percentage difference between the corresponding muscle parameters on the left and right sides

Bold values indicate *p* < 0.05

*CSA* cross-sectional area, *PDFF* proton density fat fraction, *LDH* lumbar disc herniation, *IQR* interquartile range, *MF* multifidus, *ES* erector spinae, *PM* psoas major, *GM* gluteus medius

### VBFF, LL, and SS

There were no significant differences in vertebral VBFF, LL, and SS in both the crude and adjusted analyses (*p* > 0.05, *d* = 0.07–0.19) (Table 5).

**Table 5:** Crude and adjusted comparisons of vertebral BMFF, LL and SS between the two groups

	LDH (*n* = 85), mean (SD)	Control (*n* = 48), mean (SD)	*d*	*p* Crude	*p* Adjusted
L3_BMFF (%)_	37.8 ± 8.8	36.8 ± 14.2	0.08	0.618	0.927
L4_BMFF (%)_	38.8 ± 9.3	37.5 ± 14.7	0.11	0.529	0.805
L5_BMFF (%)_	40.2 ± 10.1	38.2 ± 12.9	0.17	0.308	0.557
LL	23.7 ± 9.7	25.4 ± 7.8	0.19	0.300	0.097
SS	32.7 ± 7.6	33.2 ± 6.9	0.07	0.711	0.187

*BMFF* bone marrow fat fraction, *LL* lumbar lordosis, *SS* sacral slope, *LDH* lumbar disc herniation, *SD* standard deviation

### Subgroup analysis

Participants were categorised by herniation subtype into bulging (*n* = 9), protrusion (*n* = 51), extrusion (*n* = 24), and sequestration (*n* = 1). Due to the limited sample size in the sequestration subgroup, it was excluded from further statistical analysis. No statistically significant differences in muscle PDFF or CSA were found among patients with bulging, protrusion, or extrusion (all *p* > 0.05) (Supplementary Table 3).

## Discussion

While previous studies have primarily focused on the effects of individual lumbar or pelvic muscles, this present study offers a comprehensive, quantitative, and noninvasive assessment of the paraspinal and pelvic muscles in both LDH and non-LDH subjects. The findings indicated that although there were no significant differences in CSA of the muscles between the two groups, the PDFF was significantly higher in the LDH group for nearly all muscles examined. Recognition of muscle degeneration in both paraspinal and pelvic regions may provide insights into the musculoskeletal changes associated with LDH and inform rehabilitation strategies, although the directionality of these associations remains unclear.

Muscle degeneration is characterised by a reduction in size and increased fat and interstitial connective tissue, resulting in a decrease in CSA and an increase in fat content, as observed on imaging [33–35]. In this study, we primarily observed increased fat infiltration in the paraspinal muscles and GM in patients with LDH, while CSA changes were not significant. Our findings are consistent with those of Cheng et al, who reported a strong correlation between fat infiltration in the paraspinal muscles and lumbar disc degeneration, whereas no such correlation was observed for CSA [30]. One study also used fat infiltration in the paraspinal muscles as a predictor for disc degeneration [36]. It has been suggested that muscle fat infiltration is a more reliable indicator for assessing muscle degeneration and its association with spinal degeneration and clinical symptoms [37, 38]. The higher sensitivity of intramuscular fat infiltration, relative to changes in CSA, may be attributed to the infiltration of adipose tissue into muscle fibres during degeneration, which, despite muscle fibre atrophy, leads to minimal macroscopic changes in muscle size [4, 39, 40]. Muellner et al found that, over a 3-year follow-up in female patients with low back pain, the CSA of the psoas muscle did not significantly change, but there was a decrease in functional CSA accompanied by an increase in fat area and fatty infiltration, which further supports this concept [29]. Therefore, PDFF is a more sensitive marker than CSA for detecting paraspinal muscle degeneration. Sollmann et al applied similar fat quantification techniques in healthy volunteers and patients with chronic low back pain, demonstrating associations between PDFF and regional muscle variation, sex, BMI, and clinical parameters [20, 41]. These studies support the utility of q-Dixon imaging in evaluating paraspinal muscle composition. However, they did not specifically address LDH or examine pelvic muscles such as the GM. In contrast, our study included both LDH patients and controls, providing a comprehensive evaluation of paraspinal and pelvic muscles across multiple lumbar levels.

Over the years, much of the research has focused on the compositional and morphological changes in the adjacent structures of the lumbar disc, such as the vertebrae and paraspinal muscles, in patients with LDH [5, 36, 42]. However, few studies have explored the effects of the pelvic muscles, and even fewer have utilised the quantitative q-Dixon technique. Cooper et al reported that the GM was weaker in patients with low back pain compared to controls or the unaffected side during physical examinations [43]. Patients with low back pain often exhibit reduced hip abductor strength and altered muscle recruitment patterns [44, 45]. As a primary hip abductor, the GM contributes to stabilising the pelvis and lumbar spine in both the frontal and transverse planes [16, 46]. Degeneration or weakness of the GM may compromise the stability of the lumbopelvic-hip complex, leading to pelvic drop, increased lateral spinal flexion, and asymmetric axial loading on the lumbar discs [15, 16, 45, 47]. In the present study, GM PDFF was significantly higher in the LDH group, while CSA remained unchanged. After adjustment, the difference in PDFF remained marginally significant (*p* = 0.08), possibly indicating early compositional changes associated with biomechanical factors.

Muscle asymmetry has been proposed as a potential correlate of lumbar disc pathology, particularly with regard to unilateral LDH [48, 49]. Long et al reported a positive association between paraspinal muscle asymmetry and pain scores, suggesting a potential link to segmental biomechanical dysfunction [50]. In the present study, no significant intergroup differences in muscle asymmetry were observed after adjustment. Although Hides et al suggested that MF CSA asymmetry exceeding 10% may indicate dysfunction [51], Niemeläinen et al observed similar degrees of asymmetry in healthy individuals without low back pain [52]. Separately, another study reported paraspinal CSA asymmetry ranging from 0% to 17% at the L4 level in asymptomatic young adults [53]. In the present study, the median CSA asymmetry ranged from 5.5% to 9.6%, and PDFF asymmetry from 10.7% to 18.0%, with no significant differences between groups. These findings suggest that such asymmetry, although present in both LDH and control groups, may not reflect pathological muscle degeneration and should be interpreted with caution.

Previous studies have identified higher vertebral BMFF levels as being associated with more severe disc degeneration [11, 54, 55]. However, Bonnheim et al reported that this association with disc T1ρ values was no longer significant after adjusting for age, sex, and BMI in patients with chronic low back pain [11]. In the present study, BMFF was slightly elevated across all vertebrae in the LDH group compared to the non-LDH group; however, the differences were not statistically significant (*p* > 0.05). This discrepancy may be attributed to differences in study design; previous studies utilised quantitative MRI techniques to evaluate disc degeneration, whereas our study focused solely on grouping participants into LDH and non-LDH categories, without accounting for subtle biochemical variations in the discs. Similarly, LL and SS were slightly lower in the LDH group, but no significant differences were found (*p* > 0.05). Lumbar disc angulation constitutes the majority of LL, and disc degeneration is known to contribute to a reduction in LL, potentially leading to anterior sagittal imbalance, which in turn increases disc stress and loading [56]. The absence of significant morphological changes in the discs observed in some LDH patients, along with the relatively small sample size, may explain the lack of significant differences in LL and SS between groups. Additionally, subgroup analysis by herniation subtype showed no meaningful differences in fat infiltration or CSA, which may also be attributed to limited subgroup sizes. Further studies with larger, longitudinal cohorts are warranted to clarify these associations.

This study has several limitations. First, being a retrospective cross-sectional study, it cannot establish a causal relationship between LDH and degeneration of the pelvic and paraspinal muscles. This relationship could be more conclusively demonstrated through a high-quality prospective cohort study. Second, the relatively small sample size in this study may have contributed to the unclear between-group differences in certain spinal characteristics (e.g., asymmetry of the muscles, vertebral BMFF, and LL). Additionally, due to the retrospective nature of this study, standardised clinical symptom scores (e.g., VAS and ODI) were not available for most participants, limiting the ability to correlate imaging findings with pain or functional impairment. Future prospective studies incorporating such assessments are warranted to enhance clinical relevance. Finally, data collection for the pelvic muscles was restricted to the GM due to limitations in the number of imaging slices. Future studies should expand the examination to include the function, morphology, and composition of other pelvic muscles to further explore the relationship between pelvic muscle alterations and LDH.

## Conclusions

Compared to CSA, the PDFF derived from the 3D q-Dixon technique serves as a more reliable indicator for assessing muscle degeneration. In patients with LDH, the MF, ES, PM, and GM exhibit greater fat infiltration than control subjects. These findings may indicate a potential association between pelvic and paraspinal muscle degeneration and the presence of LDH, underscoring the value of the q-Dixon technique in detecting early muscular changes and informing targeted rehabilitation strategies.

## Supplementary information

ELECTRONIC SUPPLEMENTARY MATERIAL

## Data Availability

With permission from Shaoxing People’s Hospital upon reasonable request, the dataset is available from the corresponding author, Dr. Dingbo Shu. Restrictions do exist, the information isn’t available to the general public.

## Abbreviations

BMFF: Bone marrow fat fraction
BMI: Body mass index
CSA: Cross-sectional area
ES: Erector spinae
GM: Gluteus medius
LDH: Lumbar disc herniation
LL: Lumbar lordosis
MF: Multifidus
MRI: Magnetic resonance imaging
PDFF: Proton density fat fraction
PM: Psoas major
SS: Sacral slope
