# Supplementary material for: Paraspinal muscles and gluteus medius fat infiltration are both associated with lumbar disc herniation
Source: Insights Imaging. 2025 Aug 14;16:176. doi: 10.1186/s13244-025-02064-9 (PMC12354410; doi:10.1186/s13244-025-02064-9)
Supplement: Supplementary file 1 — ELECTRONIC SUPPLEMENTARY MATERIAL [file 13244_2025_2064_MOESM1_ESM.pdf]

Paraspinal muscles and gluteus medius fat infiltration are both associated with lumbar disc herniation

ELECTRONIC SUPPLEMENTARY MATERIAL

Supplementary Table 1. Results of multivariate linear regression analyses for PDFF of each muscle group.

Muscle PDFF	R^2	Predictor Variable	Regression Coefficient	*p*
PML3/4	0.48	LDH status	11.46	**0.011**
		BMI	5.24	**< 0.001**
		Age	1.14	**< 0.001**
		Sex	-16.71	**< 0.001**
ESL3/4	0.43	LDH status	10.76	0.123
		BMI	6.06	**< 0.001**
		Age	1.98	**< 0.001**
		Sex	-38.32	**< 0.001**
MFL3/4	0.36	LDH status	10.76	0.223
		BMI	4.24	**0.001**
		Age	1.57	**0.002**
		Sex	-58.22	**< 0.001**
PML4/5	0.50	LDH status	10.29	**0.038**
		BMI	6.42	**< 0.001**
		Age	1.22	**< 0.001**
		Sex	-18.98	**< 0.001**
ESL4/5	0.40	LDH status	26.26	**0.031**
		BMI	6.89	**< 0.001**
		Age	4.11	**< 0.001**
		Sex	-48.40	**< 0.001**
MFL4/5	0.39	LDH status	18.39	**0.028**
		BMI	3.84	**0.002**
		Age	1.60	**< 0.001**
		Sex	-55.02	**< 0.001**
GM	0.32	LDH status	15.71	0.080
		BMI	7.67	**< 0.001**
		Age	1.66	**0.001**
		Sex	-17.05	**0.046**

The table includes regression coefficients (β), *p* values, and R^2 values for models with PDFF as the dependent variable and LDH status, BMI, age, and sex as independent variables; PDFF: proton density fat fraction; LDH: lumbar disc herniation; BMI: body mass index; MF: multifidus; ES: erector spinae; PM: psoas major; GM: gluteus medius; Bold values indicate $p < 0.05$.

Insights Imaging (2025) Wang JP, Zhao ZH, Liang D, Xu X, Shu DB.

Variable	AUC	Sensitivity	Specificity	AUC	Sensitivity	Specificity	*p*
	CSA			PDFF			
$PM_{L3/4}$	0.576	0.788	0.396	0.738	0.494	0.896	**0.016**
$ES_{L3/4}$	0.616	0.918	0.354	0.667	0.494	0.833	0.532
$MF_{L3/4}$	0.575	0.212	0.958	0.596	0.624	0.563	0.788
$PM_{L4/5}$	0.572	0.776	0.417	0.725	0.753	0.625	**0.024**
$ES_{L4/5}$	0.565	0.847	0.333	0.684	0.741	0.604	0.164
$MF_{L4/5}$	0.552	0.859	0.313	0.625	0.965	0.271	0.369
GM	0.632	0.610	0.690	0.697	0.680	0.630	0.371

Supplementary Table 2. Receiver operating characteristic (ROC) analysis comparing PDFF and CSA for LDH diagnosis across different muscle levels.

LDH: lumbar disc herniation; CSA: cross-sectional area; PDFF: proton density fat fraction; ROC: receiver operating characteristic; AUC: area under the curve; MF: multifidus; ES: erector spinae; PM: psoas major; GM: gluteus medius;
Bold values indicate $p < 0.05$.

Supplementary Table 3. Comparison of muscle CSA and PDFF across LDH subtypes

	Protrusion (n = 51)<br>mean ± SD	Extrusion (n = 24)<br>mean ± SD	Bulging (n = 9)<br>mean ± SD	*p*
CSA (cm²)				
MFL3/4	652.3 ± 149.9	655.1 ± 173.3	561.3 ± 98.9	0.291
MFL4/5	985.2 ± 188.6	969.5 ± 195.1	898.2 ± 130.7	0.430
ESL3/4	1913.9 ± 528.3	1949.7 ± 472.4	1726.3 ± 408.6	0.380
ESL4/5	1368.9 ± 393.5	1367.5 ± 290.6	1277.8 ± 241.0	0.750
PML3/4	1039.6 ± 393.9	1095.8 ± 363.5	939.7 ± 269.7	0.662
PML4/5	1270.6 ± 404.3	1430.1 ± 410.5	1179.9 ± 302.3	0.227
GM	1426.6 ± 487.9	1698.0 ± 540.4	1660.9 ± 370.7	0.084
PDFF (％)				
MFL3/4	14.4 ± 6.0	14.6 ± 6.0	15.4 ± 3.7	0.622
MFL4/5	15.5 ± 6.2	16.2 ± 6.2	16.3 ± 4.0	0.688
ESL3/4	11.6 ± 4.6	11.4 ± 5.3	12.4 ± 3.2	0.586
ESL4/5	19.3 ± 8.3	18.9 ± 7.8	21.6 ± 5.4	0.377
PML3/4	9.7 ± 2.8	9.5 ± 3.1	9.0 ± 2.4	0.720
PML4/5	10.5 ± 3.5	10.4 ± 3.2	10.9 ± 2.0	0.869
GM	15.3 ± 5.2	14.5 ± 6.7	15.1 ± 3.0	0.398

Values were calculated as the average of the parameters from both sides of the muscles; CSA: cross-sectional area; PDFF: proton density fat fraction; LDH: lumbar disc herniation; SD: standard deviation; MF: multifidus; ES: erector spinae; PM: psoas major; GM: gluteus medius.
